# VCAM-1 targeted alpha-particle therapy for early brain metastases

**DOI:** 10.1093/neuonc/noz169

**Published:** 2019-09-20

**Authors:** Aurélien Corroyer-Dulmont, Samuel Valable, Nadia Falzone, Anne-Marie Frelin-Labalme, Ole Tietz, Jérôme Toutain, Manuel Sarmiento Soto, Didier Divoux, Laurent Chazalviel, Elodie A Pérès, Nicola R Sibson, Katherine A Vallis, Myriam Bernaudin

**Affiliations:** 1 Normandie University, UNICAEN, CEA, CNRS, ISTCT/CERVOxy group, GIP CYCERON, Caen, France; 2 Grand National Heavy Ion Accelerator, Caen, France; 3 Cancer Research UK and Medical Research Council, Oxford Institute for Radiation Oncology, Department of Oncology, University of Oxford, Oxford, UK

**Keywords:** alpha-particle therapy, early brain metastases, VCAM-1

## Abstract

**Background:**

Brain metastases (BM) develop frequently in patients with breast cancer. Despite the use of external beam radiotherapy (EBRT), the average overall survival is short (6 months from diagnosis). The therapeutic challenge is to deliver molecularly targeted therapy at an early stage when relatively few metastatic tumor cells have invaded the brain. Vascular cell adhesion molecule 1 (VCAM-1), overexpressed by nearby endothelial cells during the early stages of BM development, is a promising target. The aim of this study was to investigate the therapeutic value of targeted alpha-particle radiotherapy, combining lead-212 (^212^Pb) with an anti–VCAM-1 antibody (^212^Pb-αVCAM-1).

**Methods:**

Human breast carcinoma cells that metastasize to the brain, MDA-231-Br-GFP, were injected into the left cardiac ventricle of nude mice. Twenty-one days after injection, ^212^Pb-αVCAM-1 uptake in early BM was determined in a biodistribution study and systemic/brain toxicity was evaluated. Therapeutic efficacy was assessed using MR imaging and histology. Overall survival after ^212^Pb-αVCAM-1 treatment was compared with that observed after standard EBRT.

**Results:**

^212^Pb-αVCAM-1 was taken up into early BM with a tumor/healthy brain dose deposition ratio of 6 (5.52e10^8^ and 0.92e10^8^) disintegrations per gram of BM and healthy tissue, respectively. MRI analyses showed a statistically significant reduction in metastatic burden after ^212^Pb-αVCAM-1 treatment compared with EBRT (*P* < 0.001), translating to an increase in overall survival of 29% at 40 days post prescription (*P* < 0.01). No major toxicity was observed.

**Conclusions:**

The present investigation demonstrates that ^212^Pb-αVCAM-1 specifically accumulates at sites of early BM causing tumor growth inhibition.

**Key Point**

1. Combining anti–VCAM-1 antibodies with an alpha-emitting radionuclide, ^212^Pb (^212^Pb-αVCAM-1), provides a precisely targeted treatment against early brain metastases while minimizing healthy brain tissue damage.

**Importance of the Study**

Brain metastases remain a significant challenge despite the many advances in the treatment of metastatic breast cancer. Current available treatments of BM originating from primary breast cancer are ineffective in a significant proportion of patients, because of the late stage detection. VCAM-1, in preclinical and clinical settings, has been found to be overexpressed by endothelial cells during the early stages of BM development, representing a promising therapeutic target. We report that labeling anti–VCAM-1 antibodies with an alpha-emitting radionuclide, ^212^Pb (^212^Pb-αVCAM-1), provides a precisely targeted treatment against tumor cells, while minimizing healthy brain tissue damage owing to the short range of the alpha-particles. As we previously showed that VCAM-1 is expressed in endothelial cells adjacent to early BM in patients, our new targeted alpha-particle therapy has the potential for clinical translation. In addition, we have recently developed a fully humanized anti-human VCAM-1 antibody to facilitate clinical trials of this strategy. Taken together, our results presented here indicate that radio-immunotherapy with VCAM-1 is an interesting new approach to the treatment of BM.

The local control of many types of primary cancer has improved with oncological advances in recent years, but in some cases, prolongation of survival has been associated with the eventual emergence of brain metastases (BM). The availability of sensitive methods of brain imaging has also contributed to the increasingly frequent diagnosis of BM.^1^ Breast cancer, the most common malignancy in women in developed countries, carries an approximately 20% risk of BM, and the incidence is even higher in women with human epidermal growth factor receptor 2–positive disease.^2^ Even in cases where control of the primary cancer has favorable impact on overall survival (OS), a significant proportion of patients die as a result of BM,^3^ and the presence of BM is a poor prognostic factor, with an average OS of about 6 months.^1^ In the case of multiple BM, treatment consists mainly of external radiation therapy and/or, in a minority of cases, surgery. The radiotherapy protocol used depends on multiple parameters, such as KPS, molecular features, and the number and volume of tumors and consists of either whole-brain radiotherapy (WBRT) or image-guided stereotactic radiosurgery.^4^ However, despite the limited therapeutic effect (an increase in OS of 2‒4 mo), both radiotherapy protocols may induce cognitive deterioration in the case of multiple BM.^5^ Increasing the dose of external radiotherapy in an effort to improve tumor control is therefore not currently possible. The second reason for the low OS is that BM tend to present late when tumors are well established. This reflects the fact that during the early micrometastatic stages of development, the blood–brain barrier (BBB) remains intact, thus preventing detection of BM with conventional passive contrast (gadolinium) enhanced MRI. However, treatment during the earlier stages of development is likely to confer a much greater therapeutic benefit than in the later highly aggressive stages. The pressing therapeutic challenge for BM, therefore, is the need to treat (i) at an early stage when relatively few metastatic tumor cells have invaded the brain parenchyma, and (ii) in a molecularly targeted manner to avoid healthy brain toxicity.

We have previously shown that *vascular cell adhesion molecule* 1 (VCAM-1) is upregulated on the surface of endothelial cells (in the vessel lumen) during seeding of metastatic cells to the brain and during the micrometastatic stages of development within the brain.^6,7^ We have also shown that VCAM-1 can be used to detect BM very early by targeting an MRI-detectable contrast agent to this adhesion molecule.^6,8^ We now propose that this target could be used to direct therapy specifically to the site of brain micrometastases. Targeted radionuclide therapy, in which a radiotherapeutic agent is selectively delivered to tumor cells, is an area of active research.^9^ The recent introduction of a novel bone-seeking alpha-emitter, ^223^Ra (Xofigo), for the treatment of metastatic prostate cancer has highlighted this class of radionuclides as an effective treatment for disseminated disease.^10^ Alpha emitters used for targeted alpha therapy (TAT) emit high energy alpha-particles (between 5 and 8 MeV) with associated linear energy transfer of 50–230 keV/µm to targeted cancer cells over a short range of several cell diameters (40–80 µm). For this reason, TAT has been proposed as a treatment for metastatic tumors.^11^

In a previous in silico dosimetric modeling study, we found that lead-212 (^212^Pb), a novel alpha-particle emitting radionuclide proposed for theranostic applications, when combined with an antibody against VCAM-1 would be suitable for the treatment of early BM.^12^ It was found that during the early micrometastatic stages of a breast cancer BM model, tumor cells grew co-optively around blood vessels with a maximum penetration depth of ≤47.8 μm from VCAM-1 expressing at 21 days after intracardiac injection of human breast carcinoma cells (MDA231BR-GFP). Monte Carlo simulation of radiation dose deposition showed that ^212^Pb-labeled anti–VCAM-1 antibodies would provide a therapeutic dose to tumor cells adjacent to blood vessels, without the need for antibodies to cross the BBB.

The aim of the current study, therefore, was to determine whether VCAM-1 targeted ^212^Pb (^212^Pb-αVCAM-1) can prevent or delay the development of early BM. To achieve this aim, 3 substudies were performed: (i) a biodistribution study to assess the specificity and the dosimetry of the therapeutic approach; (ii) a therapeutic efficacy study for ^212^Pb-αVCAM-1 in BM, including comparison to external beam radiotherapy (EBRT), as WBRT is the current treatment of choice for multiple BM; and (iii) a study to determine systemic and brain toxicity of ^212^Pb-αVCAM-1.

## Materials and Methods

### Cell Culture

We used the human breast carcinoma cell line that preferentially metastasizes to the brain, MDA-231-Br (kindly provided by Dr Patricia S. Steeg of the National Cancer Institute).^6,7^ Cells were grown in Dulbecco's modified Eagle's medium (Sigma-Aldrich) with 1 g/L of glucose supplemented with 2 mM glutamine (Gibco), 10% fetal calf serum (Eurobio), and 1 mg/mL penicillin/streptomycin (Sigma) at 37°C.

### Mouse Brain Metastasis Model

All animal investigations were performed under the current European directive (2010/63/EU). This study was undertaken with the permission of the regional committee on animal ethics (C2EA-54) and the French Ministry of Higher Education, Research, and Innovation (project #10083). Naval Medical Research Institute nu/nu mice (25 g, 8 wk old, female; Janvier Labs) were maintained in specific pathogen-free housing and were fed γ-irradiated laboratory food and water ad libitum (ONCOModels). Procedures were performed on mice under general anesthesia (5% isoflurane for induction, 2% for maintenance in 70%-N_2_O/30%-O_2_). Body temperature was monitored and maintained at 37.5 ± 0.5°C throughout the experiments. For the BM model, mice were placed in the supine position and 1.75e10^5^ cells in 100 µL of phosphate buffered saline (PBS)–glutamine 2 mM were injected into the left ventricle of the heart guided by ultrasound imaging (CX50, MSI-FAS). Animals were then followed periodically by MRI (7T-MRI Bruker, Cyceron imaging platform) over a 16-day period to follow BM development. Animals were then assigned to biodistribution, therapy, or toxicity substudies.

### Biodistribution Study

Twenty-one days after intracardiac injection of MDA-231-Br cells, either ^212^Pb-αVCAM-1 (1 MBq; antibody mass, 27.03 µg; injectate volume, 100 µL) or an equivalent amount of ^212^Pb–immunoglobulin G (IgG) was administered intravenously to BM-bearing animals or to control animals that had not received intracardiac MDA-231-Br cells. Animals were euthanized 2, 4, 8, 24, and 72 h after ^212^Pb-αVCAM-1 injection and 4 h after ^212^Pb-IgG, and 100 µL of blood was collected. Animals were then perfused with saline solution and organs removed to evaluate radioactivity biodistribution. The organs analyzed were brain, liver, kidneys, lungs, spleen, heart, bone, and muscle. Whole brain cryosections of 20 µm thickness were prepared for autoradiography. In this study, 4 animals were used for each time point for the 2 radio-immunoconjugates. Activity was measured for the various organs (including brain sections) at different times: 2, 4, 8, and 24 h. These measurements were fitted by

$$\begin{array}{l} A\left(t\right)=A_{0}\cdot \left(1-e^{{-}_{r}\cdot t}\right)\cdot e^{{}_{d}\cdot t} \end{array}$$ (1)

where A0 is the maximum activity, λr and λd the rise and decay constants of the activity in the organ, and t the time. The cumulated activity A was then calculated by integrating At between 0 and 24 h.

### Autoradiography

An image phosphor plate was exposed for 23 h to brain sections (prepared from samples as part of the biodistribution study) and analyzed using a Cyclone Storage Phosphor System (Perkin Elmer). The phosphor plate was calibrated by exposure to different amounts of ^212^Pb (1 Bq, 2 Bq, 4 Bq, 10 Bq) for 23 h, thus allowing image intensity to be related to the amount of radioactivity in becquerels. After autoradiography, VCAM-1 immunostaining was performed on the brain sections. Co-registration of autoradiography images and VCAM-1 staining using PMOD software allowed evaluation of ^212^Pb-αVCAM-1 and ^212^Pb-IgG uptake in healthy brain tissue and VCAM-1 positive BM at 2, 4, 8, and 24 h post-injection (p.i.).

### Therapy Study

To evaluate the therapeutic potential of ^212^Pb-αVCAM-1, five groups of mice were used. A control group consisted of animals with BM that did not receive treatment. The ^212^Pb-IgG and ^208^Pb-αVCAM-1 groups were animals with BM and were included for comparison to ^212^Pb-αVCAM-1 (^208^Pb is a nonradioactive isotope of ^212^Pb). Lastly, ^212^Pb-αVCAM-1 was compared with WBRT. Immunohistochemistry was performed to evaluate damage to DNA double-strand breaks (DSB; γH2AX) 4 h after treatment. The effect of treatment on tumor cell proliferation and vascularization was characterized 3 days after treatment by Ki67 and CD31 immunostaining. Animals were then followed periodically by MRI to evaluate the number and volume of BM and OS.

### Magnetic Resonance Imaging

All MRI procedures were performed as previously described.^13^ The diffusion parameters were obtained from diffusion-weighted spin-echo planar images (6 diffusion directions, with 6 b values: 200, 500, 750, 1000, 1500, and 2000 s/mm^2^ and 2 reference images; b≈0s/mm^2^).

### Image Processing and Analyses

BM were delineated manually on all adjacent T2-weighted slices. BM volume was calculated by multiplication of the sum of contiguous tumor surface areas by the slice thickness. Enumeration of BM was done manually using MR images. Diffusion parameters including fractional anisotropy (FA) and apparent diffusion coefficient (ADC) were obtained from maps generated using Paravision software. Mean diffusivity (MD), axial diffusivity (AD), and radial diffusivity (RD) maps were calculated from eigenvalues with MD = (λ1 + λ2 + λ3)/3, AD = λ1, and RD = (λ2 + λ3)/2^14^. Kurtosis was estimated by fitting the signal obtained at all b values according to the non-Gaussian diffusion model as follows, assuming signal levels remain high compared with background noise: S(b) = S0.exp[−bADC + (bADC)2K/6]. Kurtosis characterizes the deviation from a mono-exponential decay and is null when water Brownian motion obeys a Gaussian law. As such, K increases with the heterogeneity of the cellular environment. Image analysis was performed with ImageJ software.

### Targeted Alpha-Particle Therapy with ^212^Pb-αVCAM-1

All reagents were obtained from Sigma-Aldrich, unless otherwise stated. ^212^Pb(NO_3_)_2_ (Orano Med) was conjugated to VCAM-1 antibody (clone: M/K; Merck Millipore) using a protocol based on previous publications.^15^ Briefly, metal chelator TCMC (Macrocyclics) was conjugated in 15-fold molar excess with VCAM-1 antibody using thiocyanate (SCN) chemistry resulting in a ligand to antibody ratio of 2 as determined by an Arsenzo III spectrophotometric assay. One milligram of TCMC-αVCAM-1 was then incubated with 37 MBq of ^212^Pb(NO_3_)_2_ at 37°C in 0.15 M NH_4_OAc buffer for 30 min. Radiochemical purity was determined to be 93.5% using instant thin layer chromatography (iTLC) in 0.1 M EDTA buffer (pH 8.4). Radio-immunoconjugate was diluted in PBS before injection into animals. The tail vein intravenous injected dose was 1 MBq per mouse, similar to a previous report.^15^ The above procedure was used for the radioconjugation of ^212^Pb-TCMC-IgG and ^208^Pb-TCMC-αVCAM-1. To assess the integrity of ^212^Pb-TCMC-VCAM-1, plasma was harvested at 2, 4, 8, 24, and 72 hours p.i. and the iTLC method was used to assess the ratio of conjugated to unconjugated ^212^Pb.

### External Beam Radiation Therapy

WBRT treatment was performed using an Xrad-225Cx irradiator (PXi, Cyceron platform) (225 kV X-rays; 3.3 Gy/min). A cone beam CT image was acquired for anatomical delineation. Treatment planning was performed using SmART-Plan software (Maastro Clinic), with segmentation, targeting, and planning performed using the cone beam CT image. Animals received WBRT to a total dose of 12 Gy delivered in 3 fractions of 4 Gy on 3 consecutive days, using a 10 mm collimator.

### Immunohistochemistry

Animals were euthanized and perfused with saline solution 4 h (for inflammation and DNA DSB staining) or 3 days (for cell proliferation and vasculature staining) after ^212^Pb-αVCAM-1 treatment. Inflammation, DNA DSBs, tumor cell proliferation, pericytes, microglia, and astrocytes were evaluated using primary antibodies against VCAM-1 (5 µg/mL, SoutherBiotech), γH2AX (2 µg/mL, Abcam), Ki67 (0.35 µg/mL, Dako), CD31 (5 µg/mL, PB Bioscience), platelet derived growth factor receptor beta (PDGFRβ) (2 µg/mL, Santa-Cruz), CD68 (1 µg/mL, Merck Millipore), and glial fibrillary acidic protein (GFAP) (3 µg/mL, Dako), respectively. Immunostaining protocols were performed as previously described.^13^ Tissue sections were examined at 20x magnification for VCAM-1 and at 40x for Ki67, γH2AX, CD31, PDGFRβ, CD68, and GFAP using a Leica DMi8 microscope. BM were identified by *Hoechst* 33342 counterstaining and through green fluorescent protein expression of MDA-231-Br cells. Whole brain images were obtained using Metavue software. For quantification of VCAM-1, CD31, γH2AX, and Ki67, three slices per animal were used and vessels, foci, and nuclei were automatically counted (ImageJ). Quantitative results of γH2AX and Ki67 staining are expressed as the percentage area of biomarker expression relative to the total tumor area. Vessel diameter was quantified as previously described.^16^

### Survival Study

Following treatment, mice were followed in a survival study. Prior to the initiation of the study, we defined 40 days as an arbitrary endpoint for OS outcome based on previous studies^17^ or when maximum tumor burden was reached.

### Clonogenic Assay

For X-ray treatment, an Xrad-225Cx irradiator was used (dose rate: 1.96 Gy/min; PXi, Cyceron platform). Tumor cells were plated in 6-well plates (750 cells/well), exposed to X-rays (0, 2, 4, 6, or 8 Gy) 2 h after cell seeding and incubated for 12 days. For TAT, the same number of cells was plated, and after 4 h exposed to ^212^Pb (0, 5, 10, 20, 30, or 50 kBq). Culture medium was replaced by fresh medium and cells were incubated for 12 days to allow colony formation. Colonies were stained with 2% crystal violet (Sigma-Aldrich) diluted in 20% ethanol. Colonies were counted manually. Four biological repeats of clonogenic assays were performed with, for each experiment, 3 wells per radiation dose. SF2 (survival fraction at 2Gy) and D50 (dose corresponding for SF = 50%) were obtained from the survival curves and used to compare radiosensitivity between X-rays and TAT. In vitro irradiations were modeled using Monte Carlo simulations performed with GATE.^18^

### Toxicity Study

To evaluate the radiotoxicity of ^212^Pb-αVCAM-1 on brain microstructure, diffusion MRI was used. FA, MD, AD, and RD were quantified to characterize the white matter organization and are predicted to be impacted by radiotoxicity. Moreover, cellularity was evaluated with ADC and kurtosis, two standard non-Gaussian diffusion parameters, which would be expected to increase and decrease, respectively, in the case of radiotoxicity in normal brain.^19^ All diffusion parameters were evaluated in whole brain region of interest. Submandibular blood collection was used for platelet and white blood cell counts at 24 h prior and at various times after radio-immunoconjugate injection. Counting was performed manually after standard blood smear hematoxylin and eosin staining. At the end of the study, animals were euthanized and blood was harvested for transaminase assays. Throughout the study, the weight of animals was measured as an indicator of global toxicity.

### Statistical Analyses

All data are expressed as mean ± SD. Student's *t*-test was used to compare radiosensitivity of cells to X-rays and ^212^Pb, and one-way ANOVA followed by Tukey's post-hoc test was used to compare differences between treatment groups. Two-way ANOVA (group and time effects) followed by Tukey's post-hoc test was used to assess differences in the volume and number of BM between treatment groups. A log-rank test was used to compare survival. Statistical analyses were obtained using JMP (SAS Institute).

## Results

### Whole Body Biodistribution Reveals High Uptake of ^212^Pb-αVCAM-1 at Sites of Brain Metastases

**Fig. 1:**
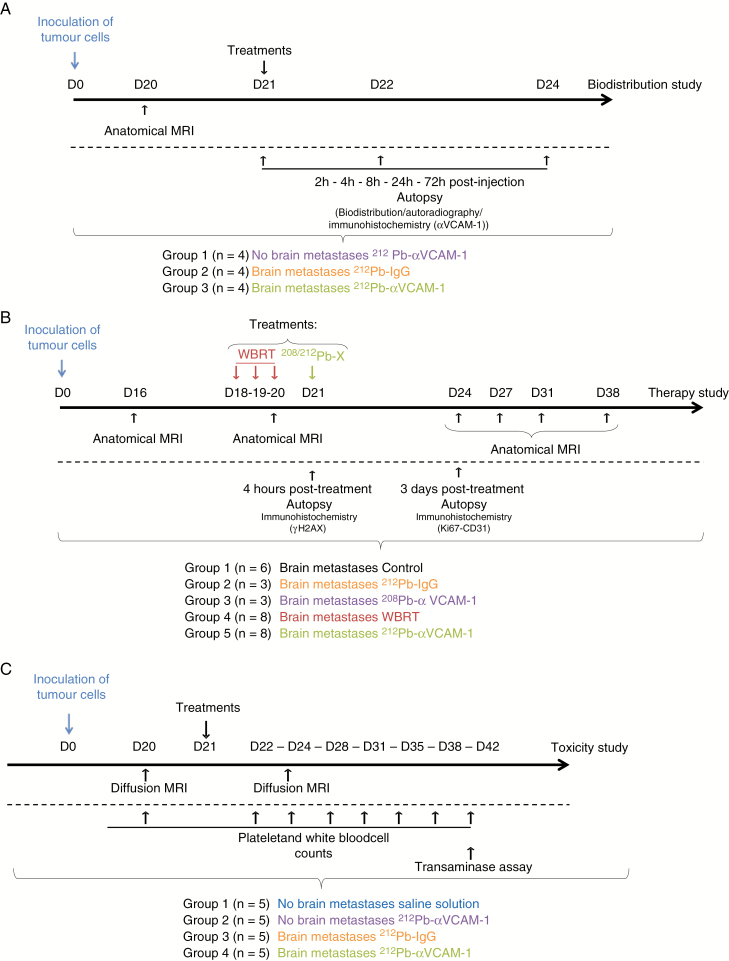
Experimental paradigm for (A) biodistribution, (B) therapy, and (C) toxicity studies.

**Fig. 2:**
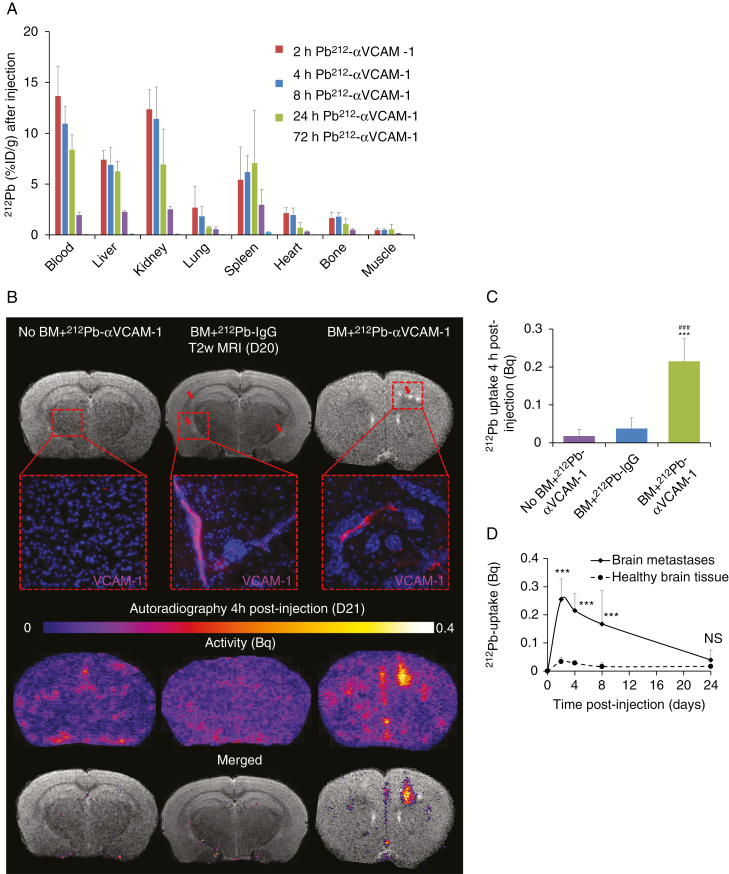
Biodistribution of ^212^Pb-αVCAM-1: whole body and brain metastases. (A) Whole body ^212^Pb-αVCAM-1 uptake biodistribution at 2, 4, 8, 24, and 72h post-injection. Mean ± SD, *n =* 4 for all time points. (B) Top: Representative T2-weighted MRI of brain from animals without tumor (sham +  ^212^Pb-αVCAM-1), with tumor with ^212^Pb-IgG/αVCAM-1 conjugated (BM +  ^212^Pb-IgG and BM +  ^212^Pb-αVCAM-1, respectively). Brain metastases appear in hyperintensity in T2-weighted (red arrows). Zoom represent VCAM-1 staining (red) in this specific healthy or BM region (blue: *Hoechst* staining). Middle: Representative autoradiography of ^212^Pb-IgG/αVCAM-1 uptake. Bottom: Merged T2-weighted MRI and thresholded autoradiography pictures (threshold: mean value of healthy tissue + 1.96*SD for *P* < 0.05). (C) ^212^Pb-αVCAM-1 brain uptake 4 h post-injection in animals without BM (sham +  ^212^Pb-αVCAM-1) or with BM with ^212^Pb-IgG/αVCAM-1 conjugated (BM +  ^212^Pb-IgG and BM +  ^212^Pb-αVCAM-1, respectively). Mean ± SD, *n =* 4 for all groups, ***P < 0.001 vs sham +  ^212^Pb-αVCAM-1 group and ^###^*P* < 0.001 vs BM +  ^212^Pb-IgG group. (D) Quantitative analyses of ^212^Pb-αVCAM-1 uptake in BM and healthy brain tissue. Mean ± SD, *n =* 4 for both groups, ***P < 0.001 vs sham +  ^212^Pb-αVCAM-1 group. NS = non-significant.

A detailed depiction of the biodistribution study, including number of animals per group, is illustrated in Fig. 1A. The stability of the ^212^Pb-αVCAM-1 complex in blood was evaluated showing that ^212^Pb-αVCAM-1 remained intact from 2 to 24 h after injection when 92.78 ± 2.19% of radioactivity present was due to ^212^Pb-TCMC-αVCAM-1. Whole body biodistribution in BM-bearing mice revealed that ^212^Pb-αVCAM-1 was predominantly present in the blood, liver, kidneys, and spleen up to 24 h p.i. (Fig. 2A). Three days after injection, ^212^Pb-αVCAM-1 retention was observed in only the spleen (0.27 ± 0.037% injected dose per gram tissue). The absorbed dose to all normal organs was <1 Gy, while blood and kidney received the highest absorbed doses of 0.84 and 0.72 Gy, respectively (Table 1). Concerning the brain, the uptake of ^212^Pb-αVCAM-1 by healthy tissue and at sites of VCAM-1 positive BM was assessed autoradiographically (Fig. 2B). Four hours after injection, no significant ^212^Pb-αVCAM-1 uptake was observed in mouse brain without metastases. Similarly, in animals with VCAM-1 positive BM that received nonspecific treatment (^212^Pb-IgG), there was no significant uptake in brain. In contrast, ^212^Pb-αVCAM-1 uptake was significantly greater (*P* < 0.001) in animals with BM (0.215 ± 0.060 Bq) following ^212^Pb-αVCAM-1 administration compared with those without BM (0.018 ± 0.014 Bq) and those with BM that received ^212^Pb-IgG (0.038 ± 0.009 Bq) (Fig. 2C). Kinetic studies (Fig. 2D) clearly revealed that the uptake of ^212^Pb-αVCAM-1 was significantly greater in BM than in healthy tissues from 2 h to 8 h p.i.; no significant difference was observed at 24 h p.i. These curves allowed us to calculate the accumulated activity in BM and healthy brain tissues and revealed a 6-fold greater accumulated activity in BM (5.52e10^8^ ± 1.41e10^8^ and 0.92e10^8^ ± 0.89e10^8^ disintegrations per gram of BM and healthy tissue, respectively).

**Table 1:** Mean organ radiation absorbed dose estimates

Organ	Mean Dose (Gy/MBq)
Blood	0.836
Bone	0.128
Brain	0.008
Heart	0.063
Kidney	0.724
Liver	0.682
Lung	0.075
Muscle	0.046
Spleen	0.781

### ^212^Pb-αVCAM-1 Reduces the Number and Volume of Brain Metastases

**Fig. 3:**
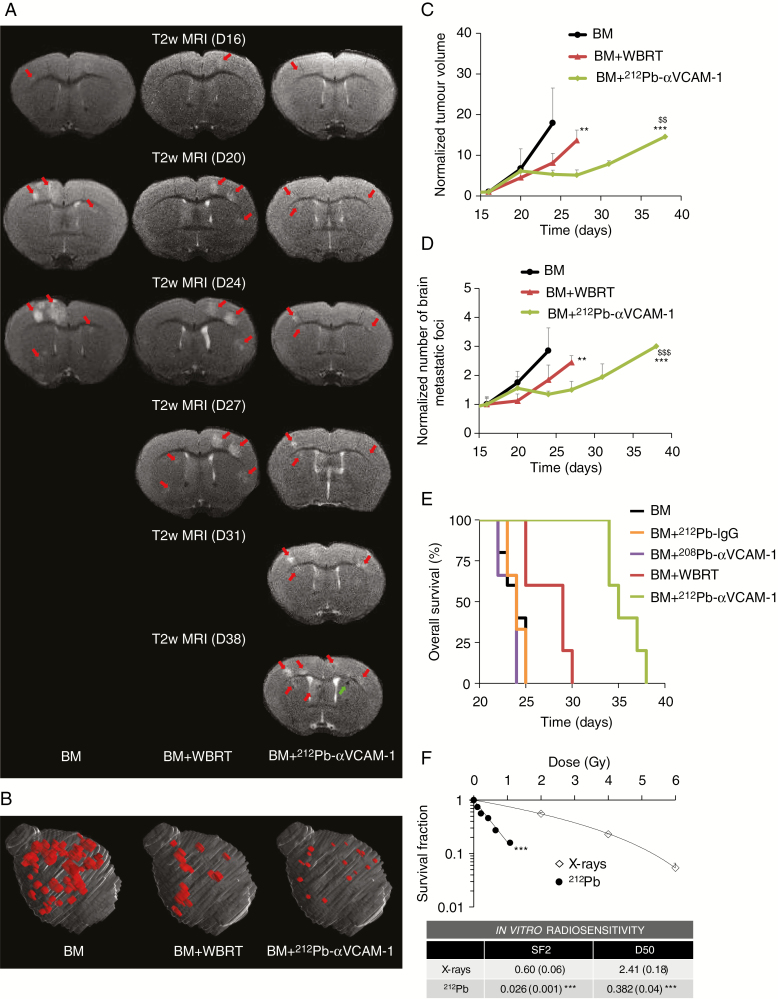
Treatment effect on number and volumes of BM and OS. (A) Representative T2-weighted MR images for the 3 treatment groups at the beginning of EBRT (D16-D17-D19) and TAT (D20) treatments at 4 time points after treatments. (B) Three-dimensional representation of BM (red) within the brain (gray) in the different groups at D24. (C) Quantitative analyses of tumor volume after treatment normalized to D16. Mean ± SD, *n =* 5 for all groups. *P* < 0.001 for time effect, **P < 0.01 and ***P < 0.001 vs BM and ^$$^*P* < 0.01 vs WBRT group. (D) Quantitative analyses of number of BM after treatment normalized to D16. Mean ± SD, *n* = 5 for all groups. *P* < 0.001 for time effect, **P < 0.01, and ***P < 0.001 vs BM and ^$$$^*P* < 0.001 vs WBRT group. (E) Kaplan–Meier curves of survival, *n =* 5 for BM, *n =* 3 for ^208^Pb-αVCAM-1, *n =* 3 for ^212^Pb-IgG, *n =* 5 for WBRT, and *n =* 5 for ^212^Pb-αVCAM-1 groups. *P* < 0.05 between WBRT and BM, ^208^Pb-αVCAM-1 and ^212^Pb-IgG and *P* < 0.01 between ^212^Pb-αVCAM-1 and all the other groups. (F) Quantitative analyses of in vitro MDA-231-Br radiosensitivity for X-rays and ^212^Pb. Mean ± SD, *n =* 4 for X-rays and ^212^Pb treatments. *P* < 0.001 for dose effect, ***P < 0.001 between ^212^Pb and X-ray treatment. SF2 = surviving fraction for 2Gy; D50 = dose that gives SF of 50%.

A detailed schedule of the therapy study is illustrated in Fig. 1B. As expected, without treatment, the BM group experienced continuous tumor growth in terms of both volume (9.05 ± 4.34 mm^3^ at 24 days) and number (40.4 ± 11.3 at 24 days) of metastases (Fig. 3A, C–D). WBRT reduced both tumor growth and the number of MRI-detectable BM in comparison to the untreated BM group (5.11 ± 1.49 mm^3^ and 32.4 ± 9.06 for tumor volume and number, respectively, at 24 days; *P* < 0.01 for both parameters; Fig. 3A, C–D). Interestingly, ^212^Pb-αVCAM-1 treatment significantly reduced tumor growth and number of BM in comparison to the control group (*P* < 0.001) and to the WBRT group (3.04 ± 0.60 mm^3^ and 18.8 ± 1.64 for tumor volume and number, respectively, at 24 days; *P* < 0.01). Treatment effects were visible as early as 3 days after the beginning of the treatment (Fig. 3B). Data cutoff for BM and BM + WBRT groups in Fig. 3B panels C and D reflects the time at which maximum tumor burden was reached.

### ^212^Pb-αVCAM-1 Increases Overall Survival

To evaluate treatment efficacy, a survival study was conducted (Fig. 3E). Animals in the untreated BM group reached the tumor volume limit at 23.8 ± 1.3 days after tumor cell injection. Animals treated with ^212^Pb-IgG and ^208^Pb-αVCAM-1 presented similar OS at 24.0 ± 1.0 days and 23.3 ± 1.3 days, respectively (no significant difference compared with untreated controls). In contrast, WBRT significantly improved OS to 27.6 ± 2.4 days (*P* < 0.05 vs control groups), while ^212^Pb-αVCAM-1 treatment further improved survival to 35.6 ± 2.1 days (*P* < 0.01 vs BM and BM + WBRT groups).

To explore the difference in efficacy of the 2 radiation modalities observed in vivo, in vitro clonogenic assays were performed. The SF2 was 0.6 ± 0.06 and 0.026 ± 0.001 for X-rays and ^212^Pb, respectively (*P* < 0.001; Fig. 3F). The D50 was 2.41 ± 0.18 and 0.382 ± 0.04 for X-rays and ^212^Pb, respectively (*P* < 0.001). The relative biological effect at 1 and 2 Gy was 4.80 and 23.08, respectively, suggesting a more pronounced radiosensitivity of MDA-231-Br cells to ^212^Pb than to X-rays.

### ^212^Pb-αVCAM-1 Decreased Cell Proliferation and Increased DNA DSBs

**Fig. 4:**
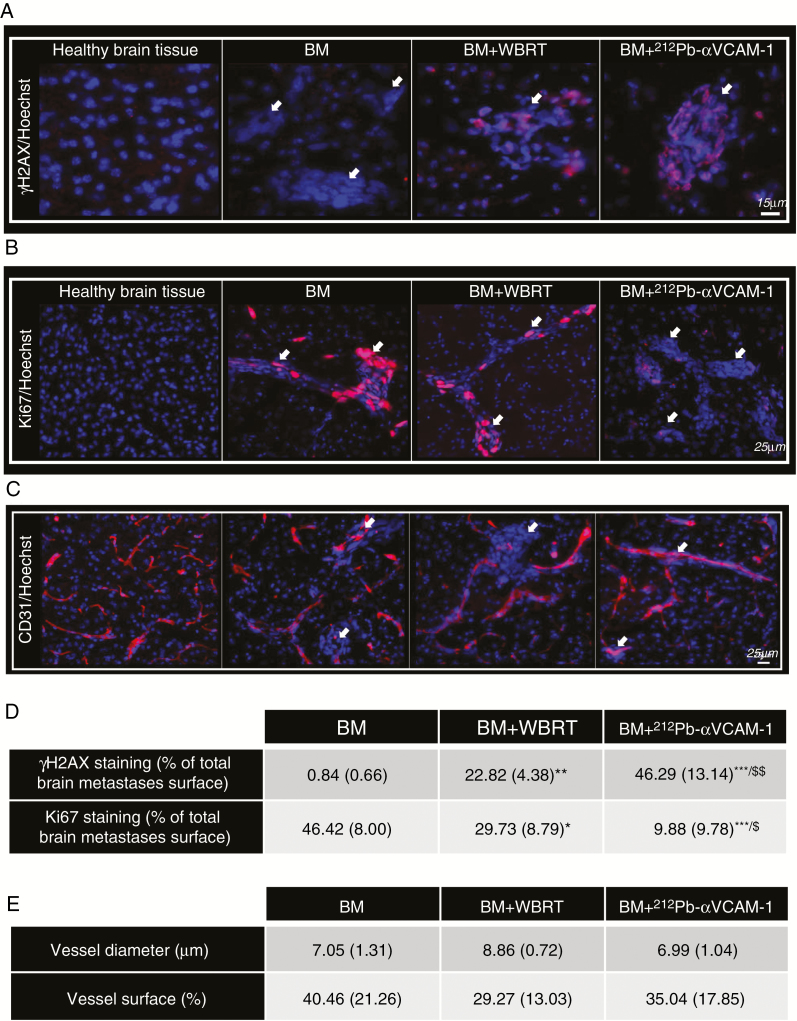
Immunohistochemical determination of treatment effect. (A) Representative images of γH2AX immunostaining (DNA DSB damage, red) with *Hoechst* 33342 nuclear counterstaining (blue). (B) Representative images of Ki67 immunostaining (cell proliferation, red) with *Hoechst* 33342 counterstaining (blue). White arrows indicate BM foci. (C) Representative images of CD31 immunostaining (endothelium, red) with *Hoechst* 33342 nuclear counterstaining (blue). (D) Data represent the mean (+SD) per group for the percentage of γH2AX and Ki67 positive cells. *P < 0.05, **P < 0.01, ***P < 0.001 vs BM group, ^$^*P* < 0.05 and ^$$^*P* < 0.01 vs WBRT group. (E) Data represent the mean (+SD) per group for vessel diameter and surface analyzed by histology.

Staining by γH2AX revealed a significant increase in DNA DSBs in BM + WBRT and BM +  ^212^Pb-αVCAM-1 groups in comparison to the BM (no treatment) group (Fig. 4A and D). Moreover, a significant difference was also observed between BM + WBRT and BM +  ^212^Pb-αVCAM-1 groups (*P* < 0.01), with a greater number of γH2AX-positive cells in the BM +  ^212^Pb-αVCAM-1 group. Staining for Ki67 demonstrated similar results, with a significant decrease in proliferation following WBRT and ^212^Pb-αVCAM-1 treatments in comparison to the BM group, and a significant difference between the BM + WBRT and BM +  ^212^Pb-αVCAM-1 groups (Fig. 4B and D).

### No Effect of ^212^Pb-αVCAM-1 on Brain Vascularization and Microenvironment

VCAM-1 is expressed on the luminal surface of endothelial cells close to early BM. The potential toxicity of ^212^Pb-αVCAM-1 treatment on brain blood vessels and the brain microenvironment was assessed immunohistochemically by CD31, PDGFRβ, CD68, and GFAP immunostaining. As expected, vessels close to the tumor (Fig. 4C) appear tortuous, disrupted, and larger in comparison to vessels in healthy tissue. However, immunostaining quantification of vessel diameter and surface did not demonstrate an effect of WBRT or ^212^Pb-αVCAM-1 treatments on vessels in comparison to the BM (no treatment) control group (Fig. 4E). From a preliminary study, we also observed that at 3 days after treatments, pericytes (detected by the PDGFRβ staining) and astrocyte activation (GFAP) do not appear to be modified whatever the treatment (Supplementary Fig. 1A–C). However, for CD68 staining, there appears to be a trend toward an increase in microglial/macrophage activation in the radiotherapy-treated groups compared with the control group (and no obvious difference between the radiotherapy modalities), but more investigations would be needed to conclude on these effects.

### Diffusion Imaging Shows that ^212^Pb-αVCAM-1 Does Not Affect Brain White Matter and Cellularity

A detailed schedule of the toxicity study is shown in Fig. 1C. As presented in Supplementary Fig. 2, the integrity of white matter was preserved after ^212^Pb-αVCAM-1 treatment compared with the control group. No significant differences were observed for diffusion metrics between the 3 animal groups (Supplementary Fig. 2E and F).

### ^212^Pb-αVCAM-1 Does Not Induce Major Systemic Toxicity

Two weeks after treatment, weight loss was: sham + saline solution = 0.85 ± 0.81 g; sham +  ^212^Pb-αVCAM-1 = 3.68 ± 1.27 g, and BM +  ^212^Pb-αVCAM-1 = 6.20 ± 3.02 g (*P* < 0.01 vs sham + saline solution group) (Supplementary Fig. 3A). To evaluate ^212^Pb-αVCAM-1 systemic toxicity, platelets and white blood cells were counted and compared with baseline. Despite a slight decrease 4 to 15 days after treatment, no significant differences were observed between groups (Supplementary Fig. 3B and C). Concerning liver toxicity, no significant changes were observed in aspartate and alanine transaminases (Supplementary Fig. 3D and E).

### Combination of ^212^Pb-αVCAM-1 and WBRT Increases Tumor Control but Dose Optimization Is Required

In a preliminary study, we investigated combining the 2 radiotherapy treatments. As shown in Supplementary Fig. 4, whereas combined treatment allows better tumor control (decrease in tumor volume and number of BM) compared with each treatment alone, it involves significant toxicity (probably as a result of too much accumulated dose), which does not allow increased OS. However, the OS is not worst than that observed with WBRT, as all the animals died about one week after treatment, as observed in the animals receiving WBRT.

## Discussion

A significant proportion of patients with controlled primary cancer die as a result of BM.^3^ Current treatment options for patients with BM from breast cancer remain limited, firstly because EBRT is performed when BM are already established and, secondly because it may induce a decline in cognitive ability for long-term survivors. Consequently, there is a critical need to develop new therapeutic approaches to target the early stages of BM.

In this study, we investigated the therapeutic potential of a targeted molecular radiotherapeutic using ^212^Pb, an alpha emitter, combined with an antibody against an early BM molecular biomarker (VCAM-1). To reflect the clinical situation as closely as possible, we used a preclinical model of BM using intracardiac injection of human metastatic breast cancer cells mimicking the invasion process observed in humans.^20^ To the best of our knowledge, this study is the first investigating the potential of TAT for the treatment of early BM. Uptake of ^212^Pb-αVCAM-1 to the region encompassing tumor cells was combined with a very low healthy brain tissue uptake, yielding a ratio of BM/healthy brain tissue dose deposition of ~6. This result is similar to findings in a clinical study targeting BM with ^124^I-radretumab for dosimetry purposes, which reported a tumor/healthy brain tissue ratio of 4.95.^21^ This 6-fold increase in accumulated activity in BM compared with healthy brain tissue could provide a better therapeutic outcome than with WBRT, where dose to normal tissue is unavoidable, leading to cognitive decline. The high uptake at sites of BM was combined with a marked increase in radiosensitivity of the human breast cancer cell line used in ^212^Pb compared with WBRT. The therapy study revealed better tumor control with ^212^Pb-αVCAM-1 in comparison to X-ray treatment, which could be ascribed to the higher radiosensitivity of cells to the high linear energy transfer of ^212^Pb compared with X-rays, leading to a greater number of irreparable DSBs.^22^ Another study, in a glioblastoma model, using an alpha emitter combined with an antibody targeting tumor vessels, ^225^Ac-E4G10, showed similar improvements in OS to the current study, but with a lower injected amount of radioactivity. The observed differences between these and the current study may reflect the longer half-life of ^225^Ac (10 days vs 10.6 h for ^212^Pb) and the BBB permeability observed in glioblastoma that is not observed in early stage BM, enabling greater alpha emitter uptake within the tumor area.^23^

With regard to systemic toxicity, although weight was significantly decreased after treatment with ^212^Pb-αVCAM-1, no blood or liver toxicity was observed. These findings are consistent with previous reports of intravenously administered ^212^Pb radio-immunotherapy.^24^

In a preliminary study we observed that ^212^Pb-αVCAM-1 had no effect on this BM microenvironment; pericytes and astrocyte activation do not appear to be modified by either form of radiotherapy. Additional studies are needed to investigate specifically the effect of both treatments on the BM microenvironment and, in particular, on inflammatory processes (eg, microglia/macrophage phenotype). We found no difference in vessel diameter or surface area. Interestingly, it has been shown in a model of glioblastoma with another alpha emitter (^225^Ac) that tumor vessel permeability and perfusion were altered,^25^ and we cannot rule this out here. For BM, a change in vessel permeability close to the tumor cells would be of interest in terms of systemic treatment, considering the constitutively low vessel permeability. This possibility warrants further investigation in future preclinical studies with more quantitative evaluation of BBB integrity using T1-weighted MRI with gadolinium DOTA contrast. In the clinic, patients with BM are treated mainly with radiotherapy, and a number of preclinical studies have highlighted the impact of external radiotherapy on the tumor microenvironment and inflammatory processes in particular.^26^ Lugade and colleagues showed an increase in VCAM-1 on B16 melanoma tumor vessels in vivo following external radiotherapy.^27^ Similarly, in the present study we found an increase in vascular VCAM-1 expression 24 h and 72 h following WBRT in tumor vessels, but not in healthy brain vessels (Supplementary Fig. 5). On the basis of our findings, we propose that external radiotherapy prior to ^212^Pb-αVCAM-1 administration could increase the specificity of our therapeutic approach by upregulating VCAM-1 locally at brain metastasis sites. This aspect highlights the importance of evaluating the impact of EBRT and targeted radionuclide therapy and the schedule of each treatment.^9,28^ Further studies are necessary with regard to the combination and the dose escalation of external radiotherapy and ^212^Pb-αVCAM-1 in a dose escalation manner, in particular with regard to balancing healthy tissue preservation and tumor control. Combining WBRT with ^212^Pb-αVCAM-1 shows great promise; however, additional studies are required to optimize the dose prescription of the combined treatments to reduce the toxicity observed in the present study

In the current study we used the MDA-231-Br cell line, a metastatic human breast carcinoma. However, further preclinical studies are necessary to determine whether the observed effects with ^212^Pb-αVCAM-1 treatment are conserved in early BM originating not only from breast cancer, but also from primary lung cancer and melanoma, which also have a propensity to spread to the brain. More generally, VCAM-1 has been shown to be highly involved in tumor growth in a number of different cancers, including melanoma, leukemia, renal, osteosarcoma, and gastric cancers, as well as downstream metastatic processes, further increasing the potential scope of ^212^Pb-αVCAM-1 therapy.^29^ Finally, the promising results generated using the MDA-231-Br model should be supplemented by similar studies in other tractable models of BM, especially in a syngeneic model.

In conclusion, we have demonstrated the therapeutic efficacy of a new TAT for early stage treatment in a preclinical model of breast cancer BM, with this first proof-in-principle investigation. ^212^Pb-αVCAM-1 accumulated selectively at sites of BM resulting in a favorable metastasis/healthy brain tissue radiation absorbed dose ratio. Moreover, ^212^Pb-αVCAM-1 uptake was associated with a significant therapeutic effect and no major toxicity. These findings suggest that early BM, which are normally not accessible to systemic treatment owing to the presence of the BBB, could be controlled using this novel therapeutic approach.

## Supplementary Material

noz169_suppl_Supplementary_Figure_1

noz169_suppl_Supplementary_Figure_2

noz169_suppl_Supplementary_Figure_3

noz169_suppl_Supplementary_Figure_4

noz169_suppl_Supplementary_Figure_5

noz169_suppl_Supplementary_Figure_6

noz169_suppl_Supplementary_Figure_7

noz169_suppl_Supplementary_Figure_8

noz169_suppl_Supplementary_Figure_Legends
