# Supplementary figures and images for: VCAM-1 targeted alpha-particle therapy for early brain metastases
Source: Neuro Oncol. 2019 Sep 20;22(3):357–68. doi: 10.1093/neuonc/noz169 (PMC7162423; doi:10.1093/neuonc/noz169)

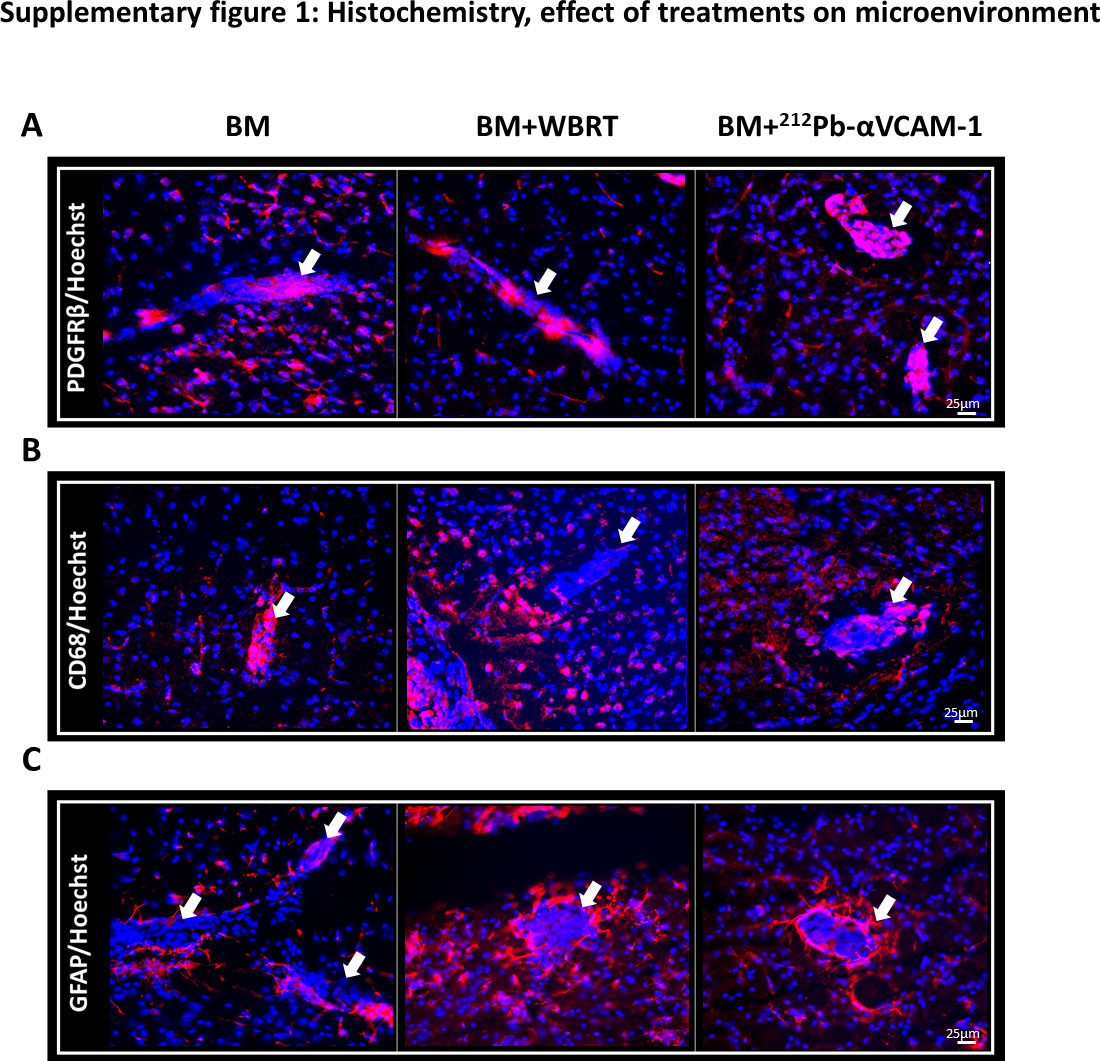

Supplement: noz169_suppl_Supplementary_Figure_1 [file noz169_suppl_supplementary_figure_1.jpeg]

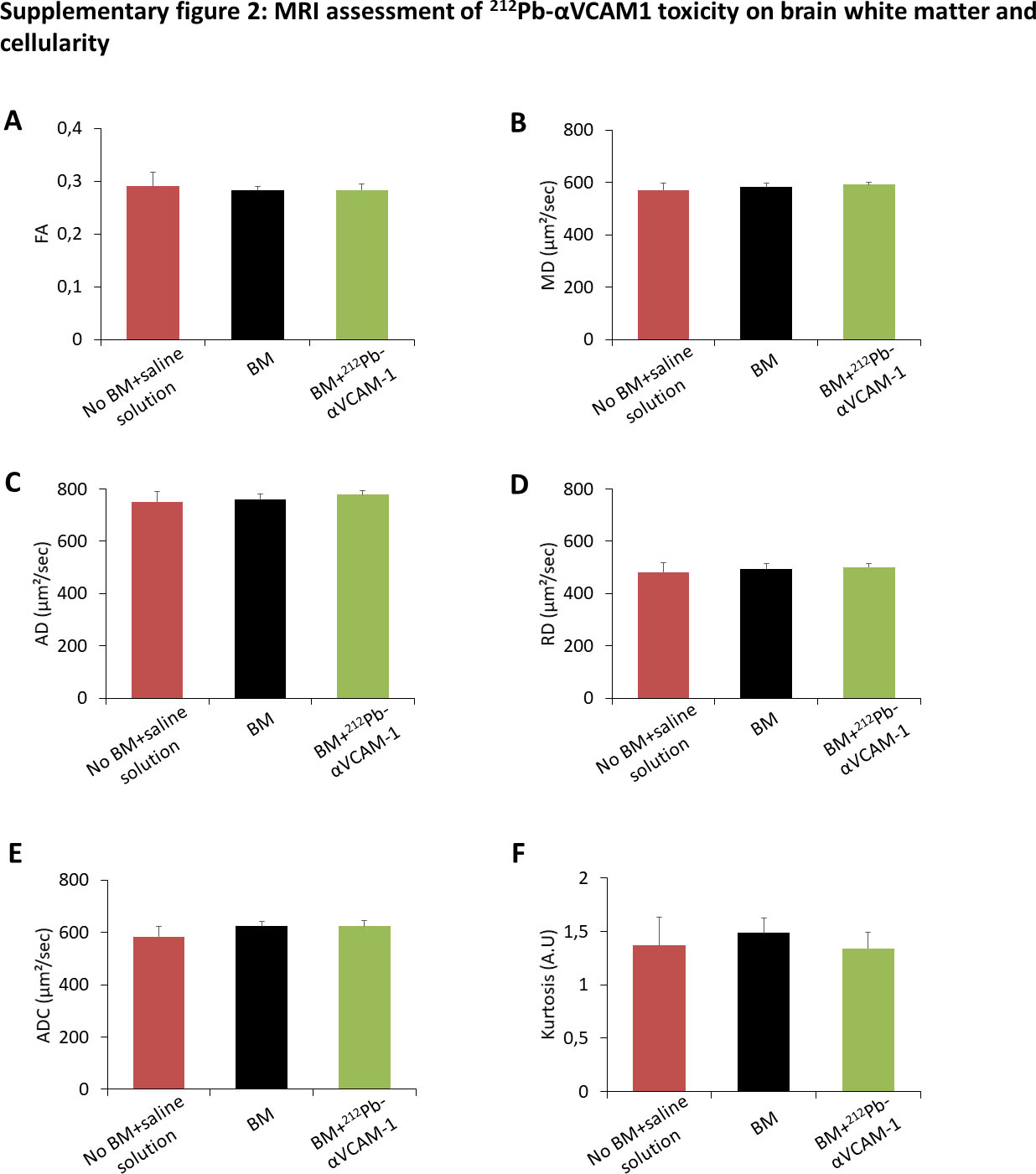

Supplement: noz169_suppl_Supplementary_Figure_2 [file noz169_suppl_supplementary_figure_2.jpeg]

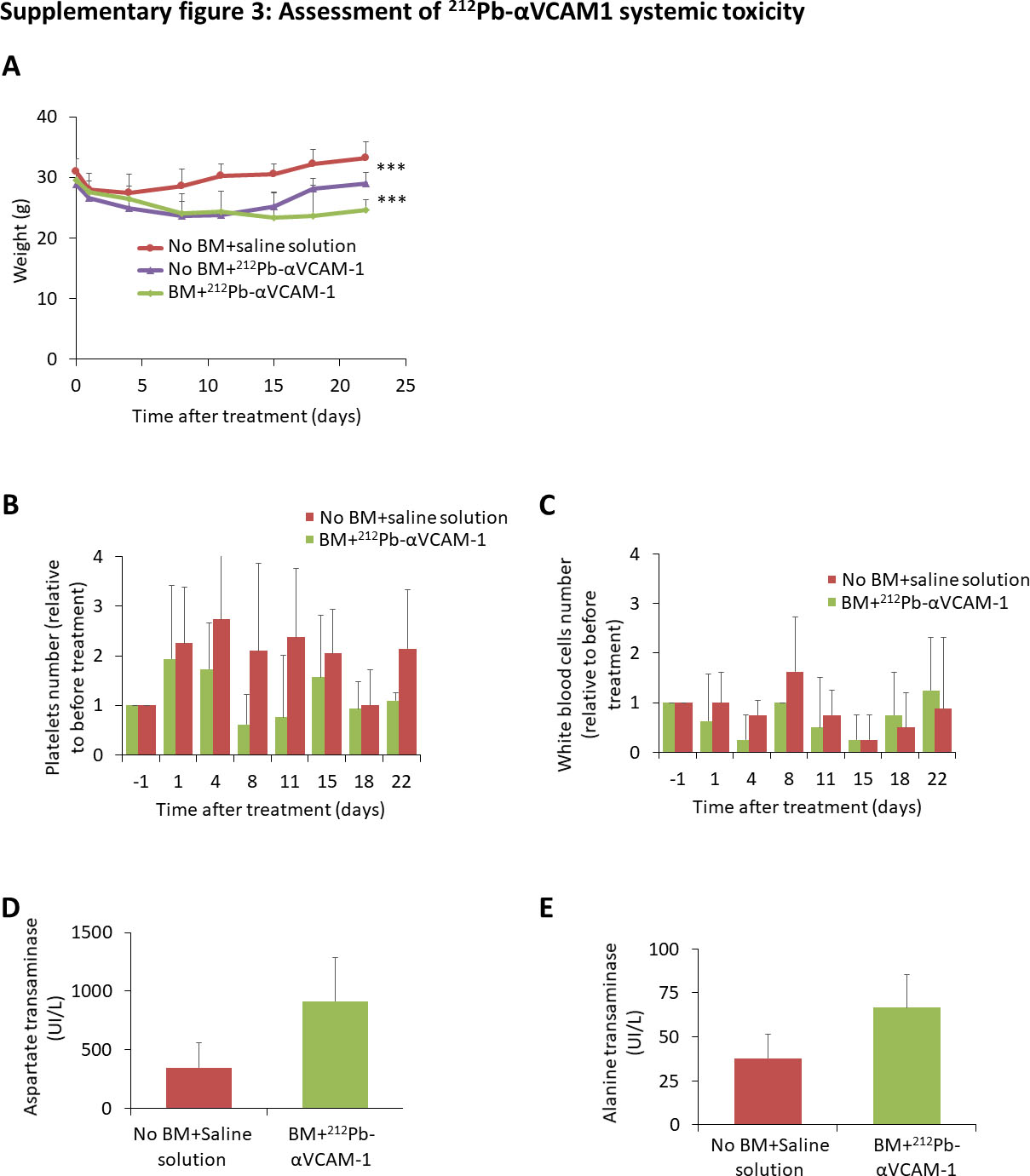

Supplement: noz169_suppl_Supplementary_Figure_3 [file noz169_suppl_supplementary_figure_3.jpeg]

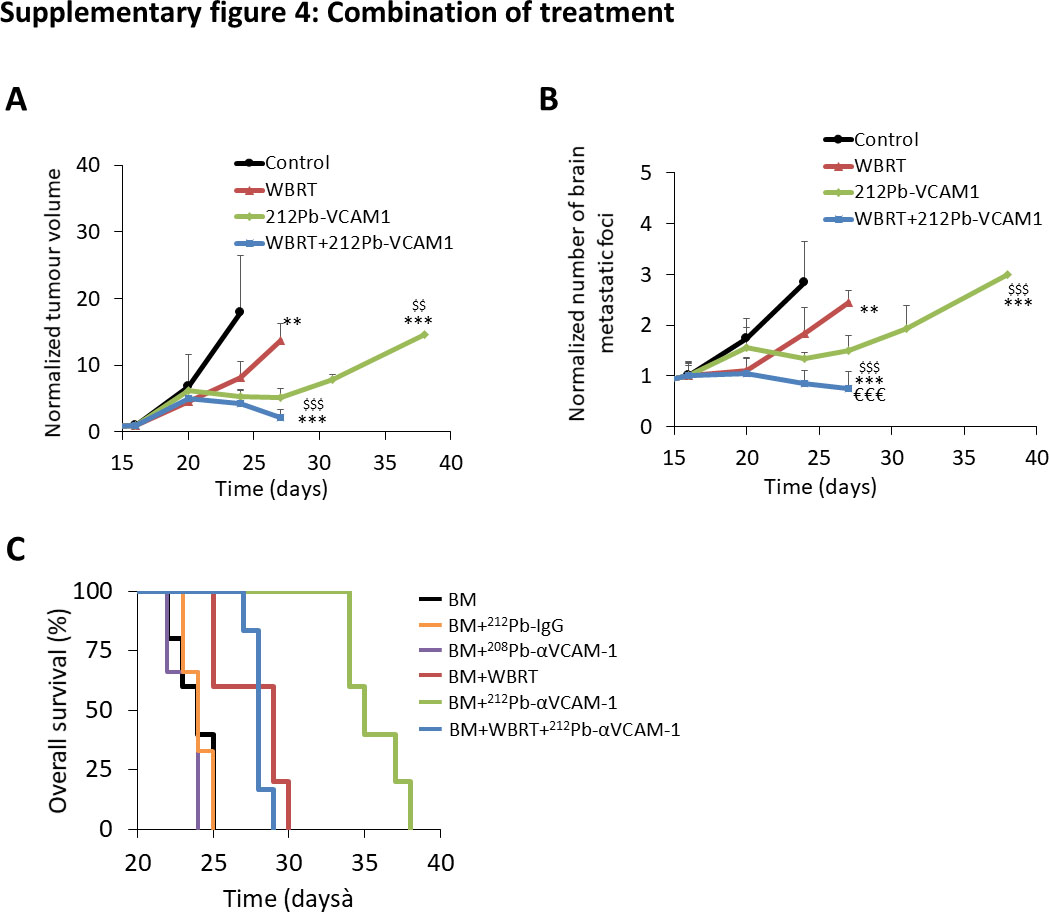

Supplement: noz169_suppl_Supplementary_Figure_4 [file noz169_suppl_supplementary_figure_4.jpeg]

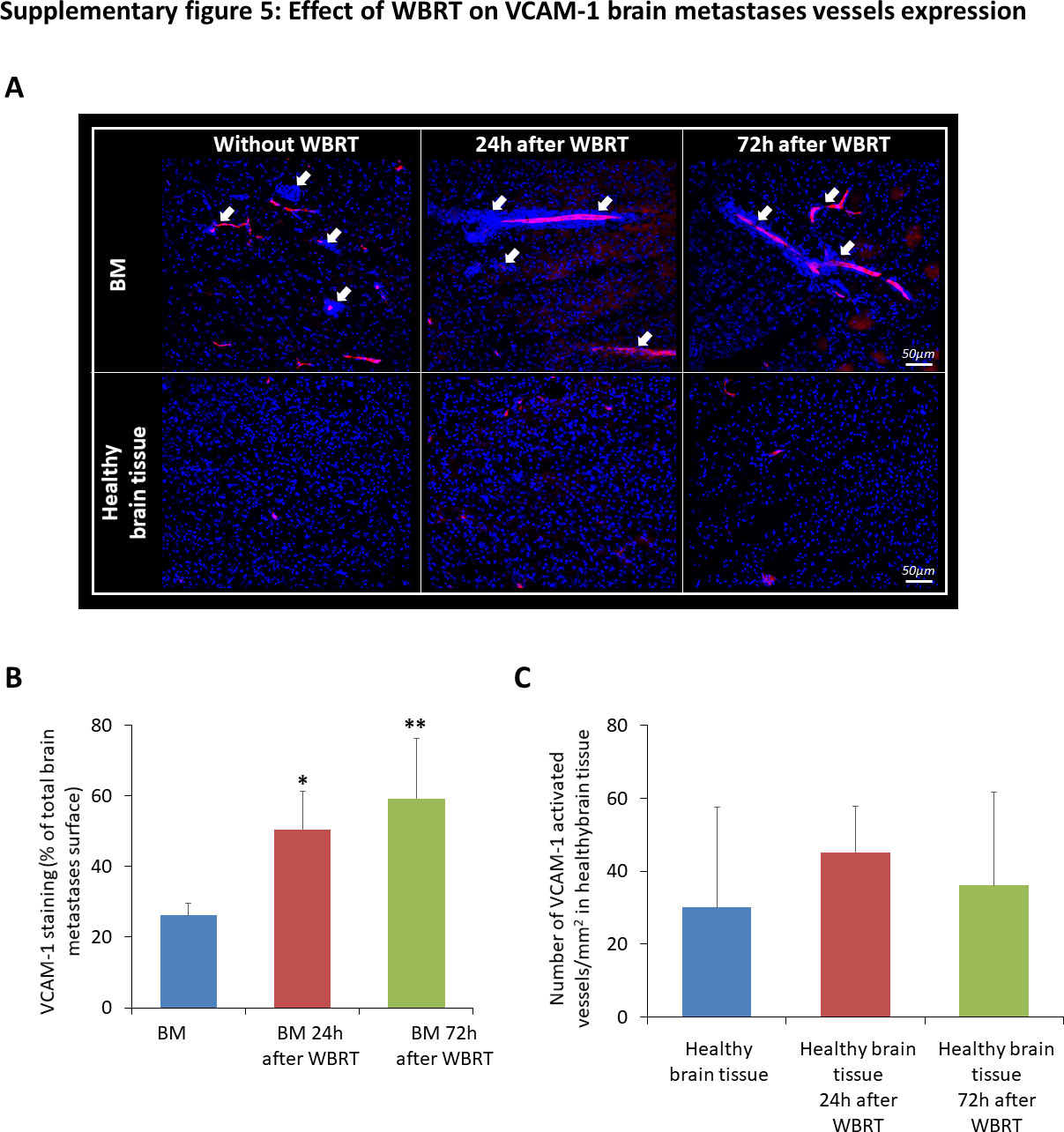

Supplement: noz169_suppl_Supplementary_Figure_5 [file noz169_suppl_supplementary_figure_5.jpeg]

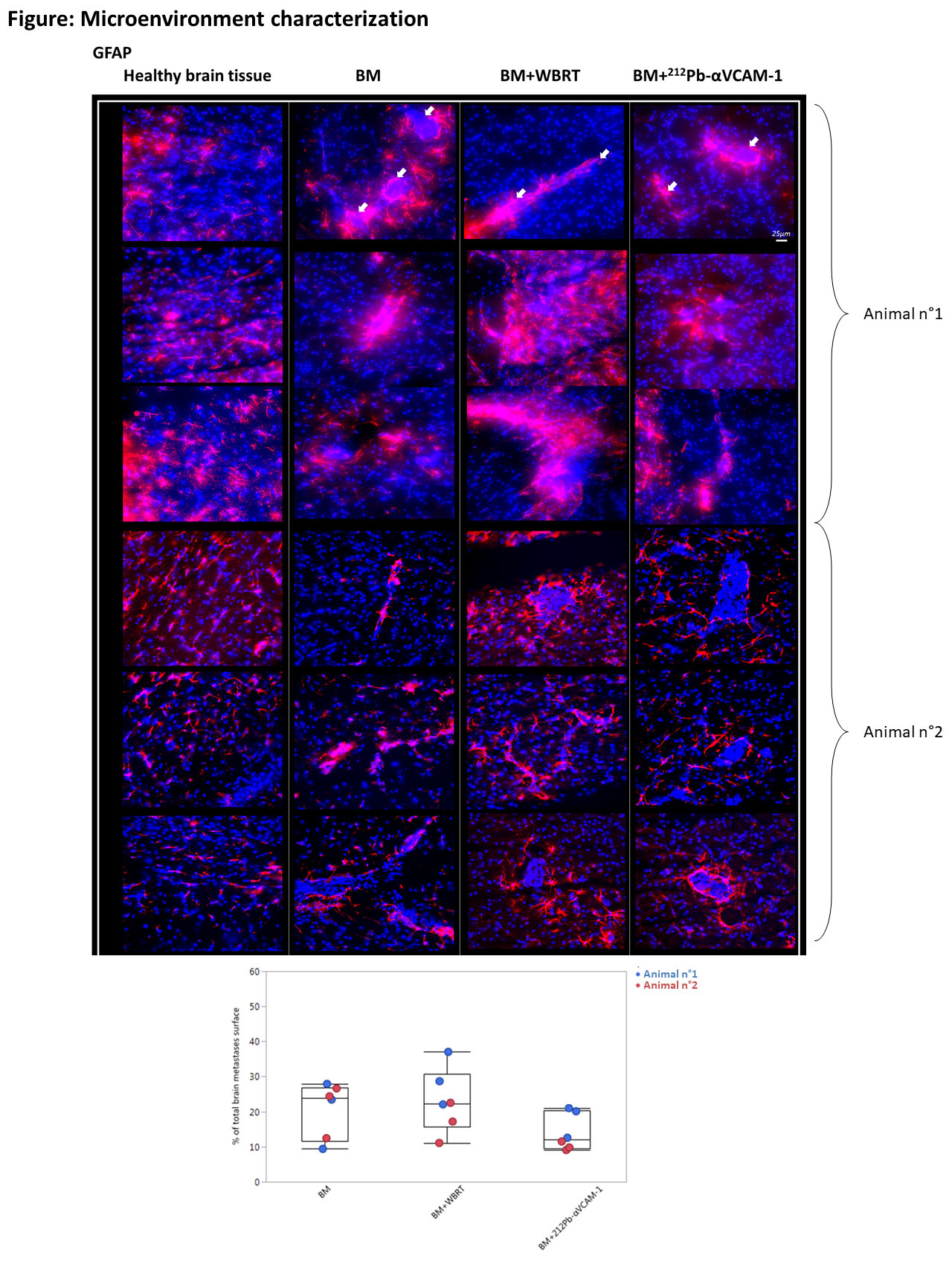

Supplement: noz169_suppl_Supplementary_Figure_6 [file noz169_suppl_supplementary_figure_6.jpeg]

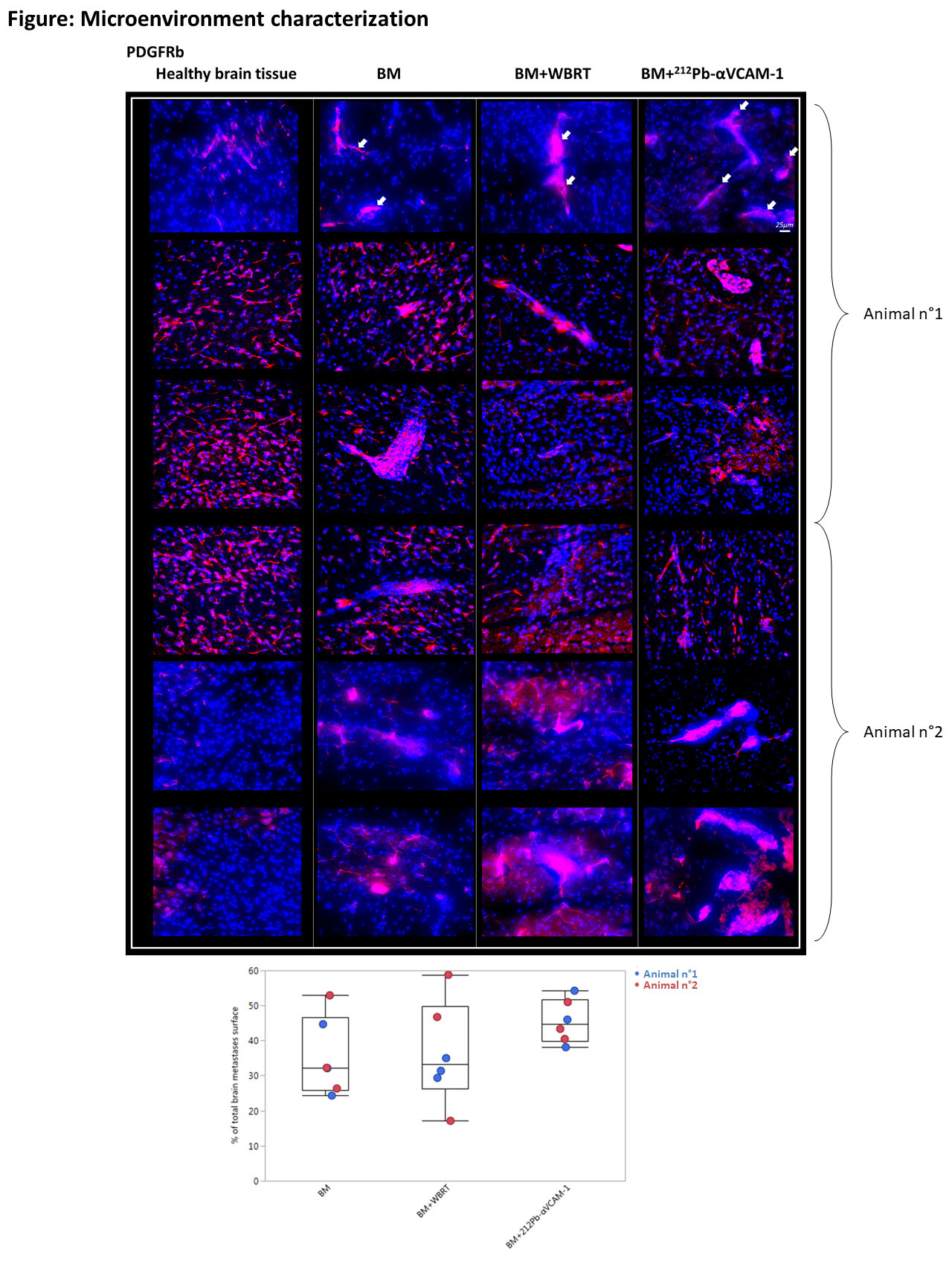

Supplement: noz169_suppl_Supplementary_Figure_7 [file noz169_suppl_supplementary_figure_7.jpeg]

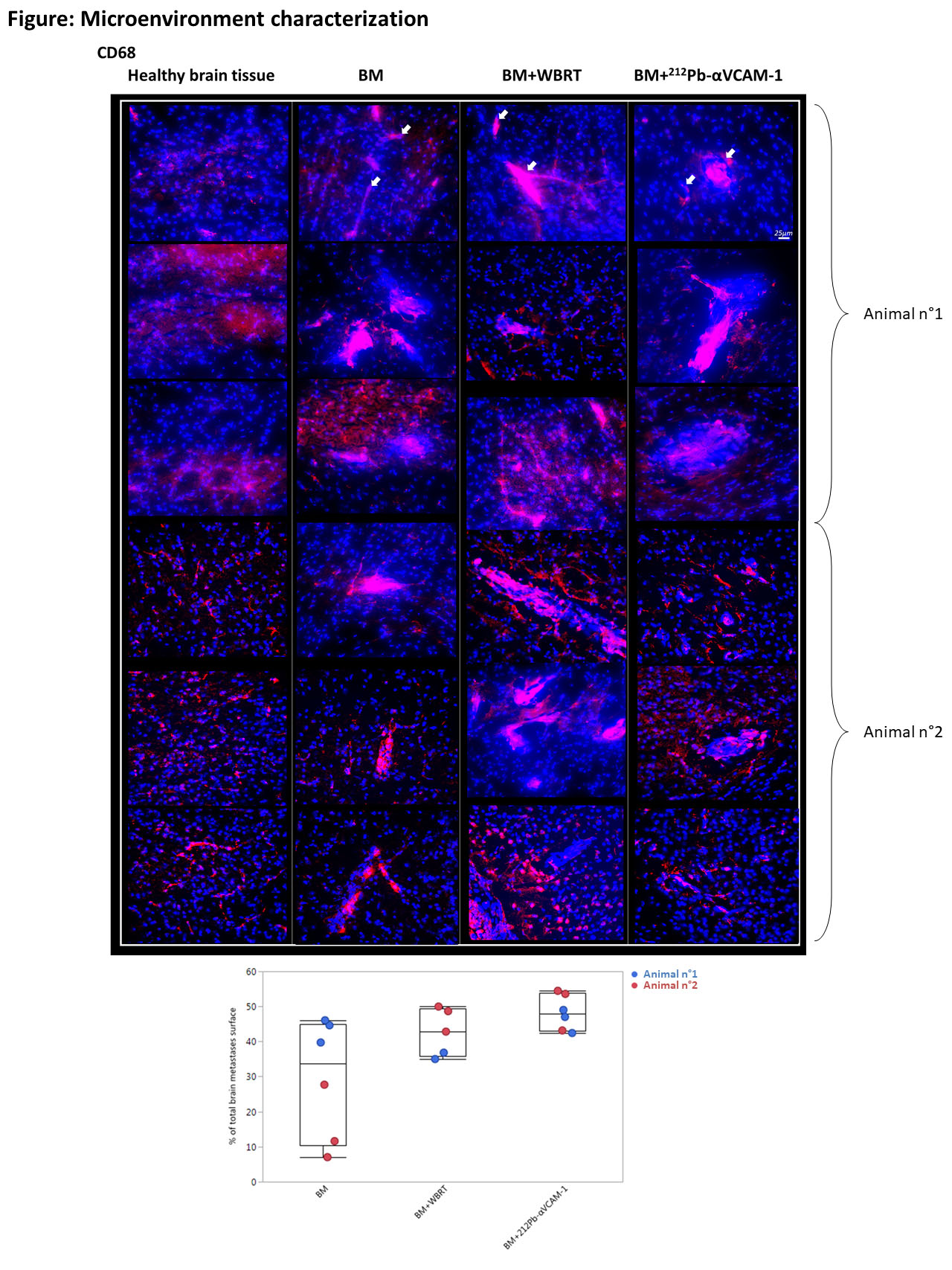

Supplement: noz169_suppl_Supplementary_Figure_8 [file noz169_suppl_supplementary_figure_8.jpeg]
