# Supplementary material for: VCAM-1 targeted alpha-particle therapy for early brain metastases
Source: Neuro Oncol. 2019 Sep 20;22(3):357–68. doi: 10.1093/neuonc/noz169 (PMC7162423; doi:10.1093/neuonc/noz169)
Supplement: noz169_suppl_Supplementary_Figure_Legends [file noz169_suppl_supplementary_figure_legends.docx]

**Supplementary figure 1: Histochemistry, effect of treatments on microenvironment**

(**A**) Representative images of PDGFRβ immunostaining (pericytes, red) with Hoescht 33342 counterstaining (blue). (B) Representative images of CD68 immunostaining (microglia/macrophages, red) with Hoescht 33342 counterstaining (blue). (C) Representative images of GFAP immunostaining (astrocytes, red) with Hoescht 33342 counterstaining (blue). White arrows indicate brain metastasis foci.

**Supplementary figure 2: MRI assessment of ^212^Pb-αVCAM-1 effect on brain white matter and cellularity**

Quantitative analyses of diffusion biomarkers of animals brain without tumour and treatment (Sham+saline solution), with tumour and with or without ^212^Pb-αVCAM-1 treatment (BM or BM+^212^Pb-αVCAM-1 respectively). Diffusion biomarkers analysed were: fractional anisotropy (A), mean diffusivity (B), axial diffusivity (C), radial diffusivity (D) apparent diffusion coefficient (E) and kurtosis (F). Mean±SD, n=3 for all groups.

**Supplementary figure 3: Assessment of ^212^Pb-αVCAM-1 systemic toxicity**

(**A**) Quantitative analyses of weight of animals without BM without treatment (Sham+saline solution), without BM and with ^212^Pb-αVCAM-1 treatment (Sham+^212^Pb-VCAM-1) and with BM and ^212^Pb-αVCAM-1 treatment (BM+^212^Pb-αVCAM-1). Mean±SD, n=5 for all groups. ***p<0.001 vs Sham+saline solution group.

Quantitative analyses of toxicity of ^212^Pb-αVCAM-1 on (B) platelets and (**C**) WBC number relative to before treatment for animals without BM and without treatment (Sham+saline solution) and animals with BM and ^212^Pb-αVCAM-1 treatment (BM+^212^Pb-αVCAM-1). Mean±SD, n=5 for both groups. Quantitative analyses of hepatic toxicity on (**D**) aspartate transaminase and (**E**) alanine transaminase. Mean±SD, n=3 for both groups.

**Supplementary figure 4: Combination of treatment on number and volumes of brain metastases and overall survival**

(A) Quantitative analyses of tumour volume after treatment normalized to D16. Mean ± SD, n=5 for all groups. p<0.001 for time effect, **p<0.01 and ***p<0.001 vs BM and $$p<0.01 and $$$p<0.001 vs WBRT group. (B) Quantitative analyses of number of brain metastases after treatment normalized to D16. Mean ± SD, n=5 for all groups. p<0.001 for time effect, **p<0.01 and ***p<0.001 vs BM, $$$p<0.001 vs WBRT group and €€€p<0.001 vs WBRT+212Pb-αVCAM-1 group. (E) Kaplan-Meier curves of survival, n=5 for BM, n=3 for 208Pb-αVCAM-1, n=3 for 212Pb-IgG, n=5 for WBRT and n=5 for 212Pb-αVCAM-1 groups. p<0.05 between WBRT and BM, 208Pb-αVCAM-1 and 212Pb-IgG groups, p<0.01 between WBRT+212Pb-αVCAM-1 and BM, 208Pb-αVCAM-1 and 212Pb-IgG groups and p<0.01 between 212Pb-αVCAM-1 and all the other groups.

**Supplementary figure 5: Effect of WBRT on VCAM-1 brain metastases vessels expression**

(**A**) Representative images of VCAM-1 immunostaining in red with Hoescht 33342 nuclear counterstaining (blue) in BM (Top) or healthy brain tissue (Bottom) without (left part), 24h after WBRT (right) or 72h after WBRT (left part). (**B**) Quantitative analyses of VCAM-1 expression on tumour vessels without WBRT, 24h or 72h after WBRT. Mean±SD, n=4 for all groups. *p<0.05 and **p<0.01 vs BM group. (**C**) Quantitative analyses of VCAM-1 expression on healthy brain vessels without WBRT, 24h or 72h after WBRT. Mean±SD, n=4 for all groups. White arrows indicate brain metastases.
